# Peer Review of “Advancing Early Detection of Major Depressive Disorder Using Multisite Functional Magnetic Resonance Imaging Data: Comparative Analysis of AI Models”

**DOI:** 10.2196/76746

**Published:** 2025-07-15

**Authors:** Anonymous

**Keywords:** major depressive disorder, machine learning, functional MRI, early detection, artificial intelligence, psychiatry

*This is a peer-review report for “Advancing Early Detection of Major Depressive Disorder Using Multisite Functional Magnetic Resonance Imaging Data: Comparative Analysis of AI Models.”*

## Round 1 Review

### General Comments

The paper [1] “Advancing Early Detection of Major Depressive Disorder: A Comparative Analysis of AI Models Using Multi-Site Functional MRI Data” examines a very relevant mental health disorder. The purpose of this paper is to identify the best artificial intelligence (AI) model for predicting early detection using a more comprehensive and versatile dataset. The paper’s contribution to psychiatry could be to provide the best AI model with specific features that can be generalized to a larger population. The paper also included a comparison of health control measures, which could improve the prediction’s accuracy. The manuscript’s most notable feature is the inclusion of 2-year longitudinal data for the early detection of major depressive disorder (MDD).

### Major Comments

1. The manuscript’s goal is to provide early but accurate detection of MDD to help with diagnosis. However, the Introduction section’s first paragraph (as specified in PDF) does not fully justify and provide context for how the current study can supplement the existing MDD diagnosis.
2. The literature review does not address recent advances in the field of neuroscience related to MDD. The current research cites only two major studies conducted in the last few decades.
3. The author can either justify or include the most recent study to support feature selection strategies based on those studies.
4. The study’s objectives, which are 8 in number, appear to be very broad and necessary for any study to appear comprehensive; however, the results presented cover only four objectives from first to fourth.
5. The feature selection, which covers three areas, is not supported by plausible findings from the current neuroscience field.
6. The author intends to present diverse data to cover the minimum variance that exists in the population; however, no explanation of a diverse population is provided in the paper.
7. The literature review presented in the manuscript could be more rigorous, first explaining the gaps in the current literature regarding the use of machine learning and deep neural networks in the detection of MDD, then explaining the best feature and detection method for MDD, and finally explaining the findings.
8. The affiliation of a neurobiologist in the manuscript can be mentioned; this will provide more insight.
9. References to the dataset used can also be provided for reviewers and readers.

## Abbreviations

AI: artificial intelligence
MDD: major depressive disorder
